# I forgot that I forgot: PTSD symptom severity in a general population correlates with everyday diary-recorded prospective memory failures

**DOI:** 10.3758/s13421-023-01400-y

**Published:** 2023-02-22

**Authors:** Taylor L. Swain, Catherine A. Keeping, Sarah Lewitzka, Melanie K. T. Takarangi

**Affiliations:** College of Education, Psychology and Social Work, Flinders University, GPO Box 2100, Adelaide, SA 5001 Australia

**Keywords:** Prospective memory, PTSD, Memory, Metamemory, Diary-recording

## Abstract

Extant research suggests a complex relationship between prospective memory (PM) and post-traumatic stress disorder (PTSD) symptom severity. In a general population, this relationship exists for self-report assessment but not objective, in-lab PM performance (e.g., pressing a certain key at a particular time, or when particular words appear). However, both these measurement methods have limitations. Objective, in-lab PM tasks might not represent typical everyday performance, while self-report measurement might be biased by metacognitive beliefs. Thus, we used a naturalistic diary paradigm to answer the overarching question: are PTSD symptoms associated with PM failures in everyday life? We found a small positive correlation between diary-recorded PM errors and PTSD symptom severity (*r* = .21). Time-based tasks (i.e., intentions completed at a particular time, or after a specified time has elapsed; *r* = .29), but not event-based tasks (i.e., intentions completed in response to an environmental cue; *r* = .08), correlated with PTSD symptoms. Moreover, although diary-recorded and self-report PM correlated, we did not replicate the finding that metacognitive beliefs underpin the PM-PTSD relationship. These results suggest that metacognitive beliefs might be particularly important for self-report PM only.

**Supplementary Information:**

The online version contains supplementary material available at 10.3758/s13421-023-01400-y.

## Introduction

Imagine a person with post-traumatic stress disorder (PTSD) forgets to: attend their therapy appointments, fill their medication prescription, and take that medication. Their symptoms worsen. They then forget to: contact their social supports at agreed times and submit work tasks by agreed deadlines. These instances of forgetting to complete future intentions are examples of prospective memory (PM) failures (Loftus, 1971). Such failures might occur in the everyday lives of people with PTSD, and consequently contribute to symptom maintenance or worsening. Existing research suggests a complex relationship between PM and PTSD symptoms; in a general population, people with greater PTSD symptom severity self-report making more everyday PM errors on questionnaires, but do not actually perform worse on in-lab, objective PM tasks (e.g., pressing a certain key at a particular time, or when particular words appear; Swain & Takarangi, 2021, 2022). However, both these methods have limitations. Objective, in-lab PM tasks might not represent typical everyday performance, because the tasks (e.g., responding to unrelated digits) and the lab environments (i.e., free of competing demands) are unlike everyday environments (Kvavilashvili & Ellis, 2004). And, metacognitive beliefs (i.e., beliefs and appraisals about one’s cognition and thinking; Papageorgiou & Wells, 2001) might bias self-report questionnaires. Thus, to increase the representativeness of the PM task, whilst reducing metacognitive biases in retrospective reporting, we used a naturalistic diary paradigm to answer the question: Are PTSD symptoms associated with everyday PM failures?

### Why PTSD is unique

Before considering extant research on the PM-PTSD relationship, we first highlight why PTSD is clinically important to PM research. PTSD is labelled a disorder of “forgetting” (Ursano et al., 2007) and of “memory” (e.g., McNally, 2006). Indeed, people with PTSD often report disorganized or fragmented memories, or perceive gaps in their memory (e.g., Ehlers & Clark, 2000; Rubin et al., 2004), that their trauma memories are more severe over time (i.e., memory amplification; e.g., Oulton et al., 2016), and that they have difficulty remembering and concentrating (e.g., Solomon & Mikulincer, 2006). Although these difficulties relate to memory for the past, PTSD symptoms might also contribute to problems remembering the future.

### PM and PTSD

Research exploring the PM-PTSD relationship is limited. In clinical populations, research relies on in-lab, objective tasks. However, these findings likely depend on the type of PM task. Time-based PM tasks are intentions completed at a particular time, or after an elapsed time-period (e.g., taking medication at 9 a.m., or every 2 h; Einstein & McDaniel, 1990). Indeed, Scott et al. (2016) and Pagulayan et al. (2018) found veterans with and without PTSD performed worse on time-based tasks (e.g., ask the researcher when the session ends after 2 min whilst completing ongoing puzzles), compared to either a control group, or a non-PTSD veteran group. Similarly, using a holiday simulation, Glienke et al. (2017) found that regardless of PTSD status, veterans performed worse on time-based PM tasks (e.g., at 10:30, eat breakfast), than non-PTSD, non-military controls.

There is also evidence the PM-PTSD relationship exists for veterans with PTSD on event-based tasks – intentions that occur in response to environmental cues (e.g., taking medication with breakfast; Einstein & McDaniel, 1990). In the Glienke et al. (2017) study, veterans also performed worse in the holiday simulation on event-based tasks (e.g., after your appointment, go to the shops), compared to non-PTSD, non-military controls. Additionally, McFarland et al. (2016) found greater PTSD symptom severity among veterans correlated with poorer event-based PM performance (i.e., pressing a key in response to a word during a multiple-choice trivia task). Although Korinek et al. (2021) found no difference between groups with and without PTSD on a similar task, veterans’ PTSD symptom severity negatively correlated with event-based PM performance (i.e., greater symptom severity was related to worse PM). And Pagulayan et al. (2018) also found worse event-based PM performance among veterans with self-reported blast-related traumatic brain injury (TBI) compared to a control group.

In a non-clinical, general population sample (Mechanical Turk), participants who reported worse PTSD symptom severity also *self-reported* more PM errors on PM questionnaires (Swain & Takarangi, 2021). However, an undergraduate general population did not perform worse on in-lab, objective PM tasks involving the maintenance of intentions (i.e., pressing a certain key after 2 min, or when certain words appear) whilst they completed ongoing tasks (e.g., multiple choice trivia; Swain & Takarangi, 2022). Altogether then, the nature of the PM-PTSD relationship seems to depend on: measurement type (e.g., in-lab tasks vs. naturalistic tasks), task type (i.e., time- vs. event-based) and sample type. Here, we focus on PM measurement and task types.

### Limitations of lab-based PM

Lab-based PM tasks have two key limitations. First, they often have weak *ecological validity* (see Sugden et al., 2021, for review), because they do not investigate PM in a way that corresponds to everyday life, and thus cannot necessarily be *generalized* to everyday tasks (Kvavilashvili & Ellis, 2004). Lab environments are dissimilar to everyday environments (Kvavilashvili & Ellis, 2004), and typically focus on one primary task. In fact, the absence of competing demands requiring cognitive resources – for example, work demands, or social media – allows participants to allocate more resources to that primary task than they typically might in everyday life. Second, and relatedly, lab-based cognitive assessments – like neuropsychological evaluations – typically measure a person’s “peak” or “best” performance (Chaytor & Schmitter-Edgecombe, 2003), rather than their average performance. Objective PM measures – including the Einstein and McDaniel (1990) paradigm or the Memory for Intentions Screening Test (Raskin et al., 2010), where participants complete ongoing tasks while maintaining intentions – likely also capture maximal, rather than average, performance (e.g., Rummel & Kvavilashvili, 2019).

These two limitations of lab-based PM assessment may be particularly important for the PM-PTSD relationship. Ellis and Ashbrook (1989) propose that a person’s emotional state regulates how they allocate cognitive resources. People in a more persistent negative emotional state, like PTSD, might allocate fewer resources to everyday PM tasks. Indeed, we know that people with PTSD inefficiently allocate cognitive resources towards other tasks (e.g., working memory; Shaw et al., 2009), likely because symptom management or other competing demands occupy these resources. But perhaps in-lab – i.e., without competing demands – they may allocate cognitive resources more efficiently. Additionally, the maladaptive strategies people with PTSD often use to control their environment for ongoing threat, or to manage unpleasant symptoms such as intrusions, may be reduced in-lab, allowing for effective PM. For example, in everyday environments, people with worse PTSD symptoms may avoid trauma-related thoughts like intrusions – for example, by occupying their mind with other things (i.e., thought suppression) – resulting in fewer available resources for PM. Moreover, PTSD symptoms themselves indicate poor attentional control (i.e., inability to control intrusive thoughts and difficulty concentrating; Esterman et al., 2013). People with PTSD or worse PTSD symptoms may also avoid making future plans – to avoid encountering additional stressful or traumatic experiences. However, in-lab (i.e., a controlled, predictable environment) they may rely less on such strategies. Essentially, in-lab environments might mitigate the typical resource allocation difficulties people with PTSD experience.

### Limitations of self-report PM

Compared to lab-based tasks, PM questionnaires examine everyday errors (e.g., forgetting to post a letter, or contact a friend) that occur in typical everyday environments. However, because such tasks require participants to report *perceived* error frequency over a specific period (e.g., the past month), without providing evidence for such errors (PMQ; Hannon et al., 1990; PRMQ; Smith et al., 2000), participants are likely vulnerable to recall biases (Kihlstrom et al., 1999). Thus, participants’ reported errors may reflect metacognitive concerns about PM (e.g., “*I have a generally poor memory, so my PM is poor*”), not just actual performance (e.g., Hainselin et al., 2021).

These metacognitive beliefs might be exaggerated among people with worse PTSD symptoms. We know people with PTSD hold negative beliefs about themselves and their memory (Ehlers & Clark, 2000; Wells, 2002) and that these negative beliefs correlate with self-report PM errors (Swain & Takarangi, 2021, 2022). We also know people with PTSD demonstrate memory biases generally, including negative interpretation of ambiguous material (Bomyea & Allard, 2017) and enhanced memory for negative content (Durand et al., 2019). Therefore, people with PTSD may be particularly susceptible to perception and memory biases when self-reporting PM.

### The current study

Altogether, the limitations of these PM tasks likely obscure the PM-PTSD relationship. Thus, our aim here was to explore the relationship using an alternative assessment method – diary-recording. PM researchers have used experience-sampling techniques, for example, probing participants via text message or phone call over several days/weeks, to assess whether they were making an error, or thinking about an intention (Anderson & McDaniel, 2019; Gardner & Ascoli, 2015). Others have used retrospective questionnaires completed at the end of each day (for 5–14 days), where participants checked whether they made specific errors (e.g., “*to do an errand or chore*”; Mogle et al., 2022), or free recalled their errors (Haas et al., 2020). Few have specifically asked participants to free record all their PM errors in a diary, over a specified period (i.e., 1–7 days; Laughland & Kvavilashvili, 2018; Niedźwieńska et al., 2020). Furthermore, only one used *both* a free record diary *and* reminded participants via a watch vibration (Brazauskiene et al., 2020). Thus, here, we combined free recall and experience sampling to maximize the number of PM errors we captured. Although diary-recording still requires participants to self-record their errors, the errors are less retrospective (i.e., participants record at the time of the error, or as soon as they remember they made an error), and require participants to provide evidence of, rather than just retrospectively judge, their errors. Importantly, this diary approach mitigates the limitations of *both* questionnaires and lab-based assessment methods by: (1) increasing the ecological validity of PM tasks and the task environment, (2) capturing participants’ “average” rather than peak performance, and (3) reducing memory biases in self-reporting (i.e., requiring real-time objective examples).

### Study aims

Our primary research question was: are PTSD symptoms correlated with *diary-recorded* everyday prospective memory failures? We asked participants to record PM errors in a 4-day diary (including whether each error was time- or event-based). We prompted them three times per day to record any errors they might have forgotten. Prior to the diary, participants completed the same questionnaires (i.e., self-report PM, negative beliefs and strategies, PTSD symptom measures; and physical and mental health comorbidities that might account for a relationship between PM and PTSD symptoms: possible TBI, alcohol dependency, learning/developmental disorder, childhood trauma, and depression, anxiety, and stress symptoms) as in Swain and Takarangi (2021, 2022).

### Hypotheses

We have two competing predictions. On the one hand, if diary reporting aligns with self-report questionnaires – due to the generalizable nature of everyday PM tasks and everyday environments – we predict that diary-reported PM failures will positively correlate with PTSD symptom severity (Swain & Takarangi, 2021). We expect that the correlation will be larger for time- than event-based failures, because time-based tasks rely heavily on executive functions (e.g., monitoring; see Einstein & McDaniel, 1990; Scott et al., 2016) that are often impaired in PTSD. On the other hand, if PTSD symptoms are unrelated to actual PM performance – and only correlate with self-report PM questionnaires because of metacognitive biases in reporting, specifically amongst people with more severe PTSD symptoms (e.g., Bomyea & Allard, 2017; Durand et al., 2019) – we predict that diary-reported PM failures will *not* correlate with PTSD symptom severity (Swain & Takarangi, 2022).

Based on the idea that metacognitive beliefs and maladaptive strategies might underpin the PM-PTSD relationship – and the mediation results from prior research (e.g., Swain & Takarangi, 2021, 2022) – we also predict that cognitive confidence, negative cognitions about the self, beliefs about memory, thought suppression, and willingness to future plan, will partially mediate the relationship.

We have two secondary interests. First, given questionnaire-based, but not lab-based, PM errors correlate with everyday functioning (Swain & Takarangi, 2022), we examined the relationship between PTSD, PM and everyday functioning (e.g., social or occupational functioning). We predict that PTSD symptoms might contribute to greater PM errors, resulting in greater impairment (i.e., a mediation). Second, stress may be an important predictor in the PM-PTSD relationship (Swain & Takarangi, 2021); thus, we examined the relationship between PTSD, PM and perceived everyday stress. We predict that PTSD symptoms might contribute to greater stress, resulting in more PM errors (i.e., a mediation).

## Method

### Participants

Our final sample was 260 – based on the idea that correlations typically stabilize as they approach 260 participants (Schönbrodt & Perugini, 2013, 2018). We recruited 327 participants using varied methods: the Flinders University SONA system (credit: *n* = 108, paid: *n* = 22), social media (*n* = 136), flyers (*n* = 35), an online marketplace (i.e., Gumtree; *n* = 7), and other (e.g., referrals from others, *n* = 19). Twenty-one people completed Phase 1 and were excluded from further participation (e.g., for failing three attention checks embedded within that phase (Berinsky et al., 2014; Hauser & Schwarz, 2015), providing an invalid mobile number, or having already participated). Per our pre-registration (see https://osf.io/pgw3u), we excluded a further 41 people who completed all study phases but recorded an insufficient number of PM errors (i.e., < 2; *n* = 39), or completed the diary incorrectly (*n* = 2). We excluded one person who did not complete the surveys central to our research question (i.e., trauma measures). Four participants withdrew during the diary phase. Our sample were aged 18–69 years (*M* = 25.74, *SD* = 9.51) and identified as 15.0% male (*n* = 39), 80.8% female (*n* = 210), 3.8% non-binary (*n* = 10); one person preferred not to identify their gender (0.4%). Most participants were Caucasian (73.8%, *n* = 192), followed by Asian (13.8%, *n* = 36), mixed race (4.6%, *n* = 12), Middle Eastern (3.1%, *n* = 8), Indian (2.3%, *n* = 6), and Aboriginal, African, Hispanic, Pacific Islander and Serbian (each 0.4%, *n* = 1). Further, 1.5% completed doctoral studies as their highest qualification (*n* = 4), 7.3% Master’s studies (*n* = 19), 31.2% a Bachelor’s degree (*n* = 81), 9.6% an associate degree or certificate (*n* = 25), 49.5% senior high school or equivalent (*n* = 129), and two had only completed junior high school (0.8%). Participants were reimbursed $30 or course credit. All data can be found at: https://osf.io/wmvhb/

### Measures

#### At-home prospective memory diary

Participants completed an at-home online PM diary based on Laughland and Kvavilashvili (2018). In the diary, participants: recorded the time the error occurred, the time they realised they had made the error, described their error (i.e., what they were doing, what they forgot, where they were), and reported if the error was time- or event-based. Then participants were asked if they had any more instances to record, before finalizing their response (see Online Supplementary Materials (OSM) for full diary questions).^1^

Each diary entry counted as one PM error; therefore, each participant’s PM error score was equivalent to the total number of valid diary entries they made (i.e., after exclusions with incomplete details). Participants could also enter multiple errors subsequently, but each individual entry counted as one error.

#### Post-diary survey

To explore diary compliance and aid use, after the diary-period we asked participants if they carried their phone on every day of the study. If they responded “no”, they reported the number of days they did not carry their phone. We then asked participants to estimate the percentage of errors they recorded over the diary-period (i.e., “*please indicate the percentage of forgetting instances you were able to record out of ALL your forgetting over the diary period*”). Participants reported the ease of: carrying their phone and recording their errors (*1 = very easy, 4 = very difficult*). Finally, we asked participants (a) if they used aids to help them remember, (b) which aids they used, from a list (e.g., lists, electronic reminders), and (c) if that aid use was typical of their behaviour.

#### Questionnaires

#### Historical/physical health comorbidities

##### Learning/developmental disorder screening

We asked participants whether they had ever been: (a) diagnosed with a learning disorder (e.g., Attention-Deficit Hyperactivity Disorder (ADHD)), (b) placed in a learning/emotionally handicapped special class, or (c) told they had a learning disorder, and if so, by whom (e.g., teacher). We classified participants as having a learning disorder when they responded “yes” to questions (a) or (b), *and* listed doctor/other specialist or teacher on (c) (Swain & Takarangi, 2021, 2022).

##### HELPS Screening Tool (HELPS; Picard et al., 1991)

The HELPS is designed to screen for TBI. It includes five questions about TBI events, and the potential aftermath. “H” (Have you ever *H*it your *H*ead, or been *H*it on the *H*ead?); “E” (Were you ever seen in the *E*mergency room, hospital, or by a doctor because of an injury to your head?); “L” (Did you ever *L*ose consciousness or experience a period of being dazed or confused because of an injury to your head?); “P” (Do you experience any of these *P*roblems in your life daily since you hit your head? E.g., headaches and dizziness); “S” (Any significant *S*icknesses?). Participants are classified as having a potential TBI when they respond “yes” to at least one event, at least one medical assessment option, and report two or more chronic problems.

##### The Alcohol Use Disorders Identification Test (AUDIT; Babor et al., 2011)

The AUDIT was developed by the World Health Organization (WHO) to identify people whose alcohol consumption has become harmful to their health. The AUDIT has ten questions, here used in self-report form (e.g., ‘*how often do you have a drink containing alcohol?*’). These questions vary in format but typically use a Likert scale; higher scores indicate more harmful consumption (0, e.g., *never*, *1 or 2, to 4*, e.g., *four or more times per week, daily or almost daily*). Internal consistency was good in the current study (α = .80).

#### Prospective memory questionnaires

We acknowledge existing concerns around the validity of self-report PM questionnaires (e.g., Uttl & Kibreab, 2011). Thus, to comprehensively assess subjective PM, and to allow us to compare to prior research in the general population (Swain & Takarangi, 2021, 2022), we used two common PM questionnaires.

##### Prospective and Retrospective Memory Questionnaire (PRMQ; Smith et al., 2000)

The PRMQ provides a self-report measure of prospective and retrospective memory slips in everyday life. Here, we used only the PM scale, containing eight items (e.g., “*Do you forget to buy something you planned to buy, like a birthday card, when you go to the shop?*”). Participants responded on a 5-point Likert scale rating how often these occurrences happen to them (*1* = *Never, 5* = *Very often*). The PM subscale had good internal consistency (current study, α = .89).

##### Prospective Memory Questionnaire (PMQ, revised version; Hannon et al., 1995)

We used a modified version of the PMQ (Cuttler et al., 2016) because participants have difficulties understanding the original scale (Cuttler & Taylor, 2012; Uttl & Kibreab, 2011). Participants responded to 52 items (e.g., “*I missed appointments I had scheduled*”) using a Likert scale (1 = *Never,* 5 = *Very often*). They can select “not applicable” if the task does not apply (e.g., “*I forget to return books to the library by the due date*” if they do not borrow books; scored as a ‘0’). The scale was reliable (current study, α = .95).

#### Mediator variables

##### Beliefs About Memory Questionnaire (BAMQ; Bennett & Wells, 2010)

The BAMQ is used to measure positive (i.e., the need to have a complete trauma memory) and negative (i.e., negative interpretations of incomplete trauma memory) metacognitive beliefs about participants’ trauma memory. Here we used it to measure maladaptive beliefs about memory. Participants respond to 15 statements (e.g., “*having memory blanks means there is something seriously wrong with me*”) on a Likert scale (*1* = *Do not agree, 4* = *Agree very much*). The subscales had good internal consistency in the current study (α = .78–.89).

##### Cognitive Confidence Subscale of the Metacognitions Questionnaire (MCQ; Cartwright-Hatton & Wells, 1997)

The MCQ consists of five subscales designed to measure beliefs about worry and intrusive thoughts. Here we used the cognitive confidence subscale to measure confidence in memory ability. Participants responded to a series of six statements (e.g., “*I have a poor memory*”) on a Likert scale (*1* = *Do not agree, 4* = *Agree very much*). Higher scores indicate greater maladaptive beliefs (i.e., lower cognitive confidence). The cognitive confidence subscale had good reliability (current study, α = .85).

##### Negative Cognitions About the Self Subscale from the Posttraumatic Cognitions Inventory (PTCI; Foa et al., 1999)

The PTCI contains 36 items used to measure beliefs related to a traumatic experience. Here, we used only the negative cognitions about the self subscale. Participants rated a series of 26 statements that potentially represent their thinking (e.g., ‘*the event happened because of the way I reacted’*; *1 = Totally disagree, 7 = Totally agree).* This subscale had excellent reliability (current study, α = .96).

##### White Bear Suppression Inventory (WBSI; Muris et al., 1996; Wegner & Zanakos, 1994)

The WBSI measures people’s tendency to suppress negative thoughts. Here, we used the scale to measure the maladaptive strategy of not thinking about their nominated most traumatic/stressful event. Participants responded on a 5-point scale demonstrating their agreement (*1 = Strongly disagree, 5 = Strongly agree*) with 15 statements (e.g., “*There are things I prefer not to think about*”). Thus, higher scores indicate greater suppression. The scale had good internal validity (current study, α = .94).

##### Continuous Planning Scale (CPS; Prenda & Lachman, 2001)

We used the CPS to measure willingness to future plan. Participants rated five statements (e.g., “*I find it helpful to set goals for the near future*”) according to how well each statement represented them (4 = *A lot*, 1 = *Not at all*). Internal validity was acceptable in the current study (α = .67).

#### PTSD symptoms and current mental health comorbidity variables

##### Trauma History Screen (THS; Carlson et al., 2011) and PTSD Checklist-5 (PCL-5; Blevins et al., 2015)

Participants responded Yes/No to traumatic events they may have experienced (e.g., “*a really bad car, boat, train or airplane accident*”). If they responded yes, they reported: how many times each event(s) occurred, if any event bothered them emotionally, and described their most traumatic experience. Participants who did not endorse any of the listed events described their “most stressful event”. We used the PCL to measure self-reported PTSD symptom severity over the last month in reference to participants’ most traumatic/stressful event. Participants rated their symptoms (e.g., “*repeated, disturbing, and unwanted memories of the stressful experience*?”) on a 5-point scale (0 = *Not at all,* 4 = *Extremely*). PTSD symptoms as measured by the PCL-5 are continuous and the label “PTSD symptom severity” is in line with this construct label (i.e., the presence and frequency of symptoms that align with a diagnosis of PTSD, *not* whether participants have a PTSD diagnosis). The PCL-5 has excellent internal consistency (current study, α = .95).

##### Depression and Anxiety Symptoms (DASS-21; Lovibond & Lovibond, 1995)

The DASS-21 is a 21-item measure of depression, anxiety and stress. Participants rated how much each statement (e.g., “*I found it hard to wind down*”) applied to them over the past week (0 = *Did not apply to me at all*, 3 = *Applied to me very much or most of the time*). Scores are totalled for each subscale and place participants in severity ranges from normal to extremely severe. The DASS-21 had excellent internal consistency (current study: depression, α = .92; anxiety, α = .88; stress, α = .88).

##### Brief Inventory of Psychosocial Functioning (B-IPF; Bovin et al., 2018)

The B-IPF measures everyday functioning through seven questions – one for each impairment domain in everyday life (e.g., social activities, work and education). Participants responded to how much (*0 = Not at all, 6 = Very much*) trouble they experience in each domain of functioning over the past 30 days (e.g., “*I had trouble with my family relationships*”). The B-IPF had high internal consistency (current study, α = .72).

##### Perceived Stress Scale (PSS-10; Cohen & Williamson, 1988)

The PSS-10 is a shortened version of the PSS, designed to measure the degree to which a person perceives situations in their life as stressful, over the past month. Participants responded how often (*0 = Never*, *4 = Very often*) they felt or thought a certain way (e.g., *In the last month, how often have you felt nervous and “stressed”?*). The PSS-10 had good internal consistency (current study, α = .85).

#### Procedure

**Fig. 1:**
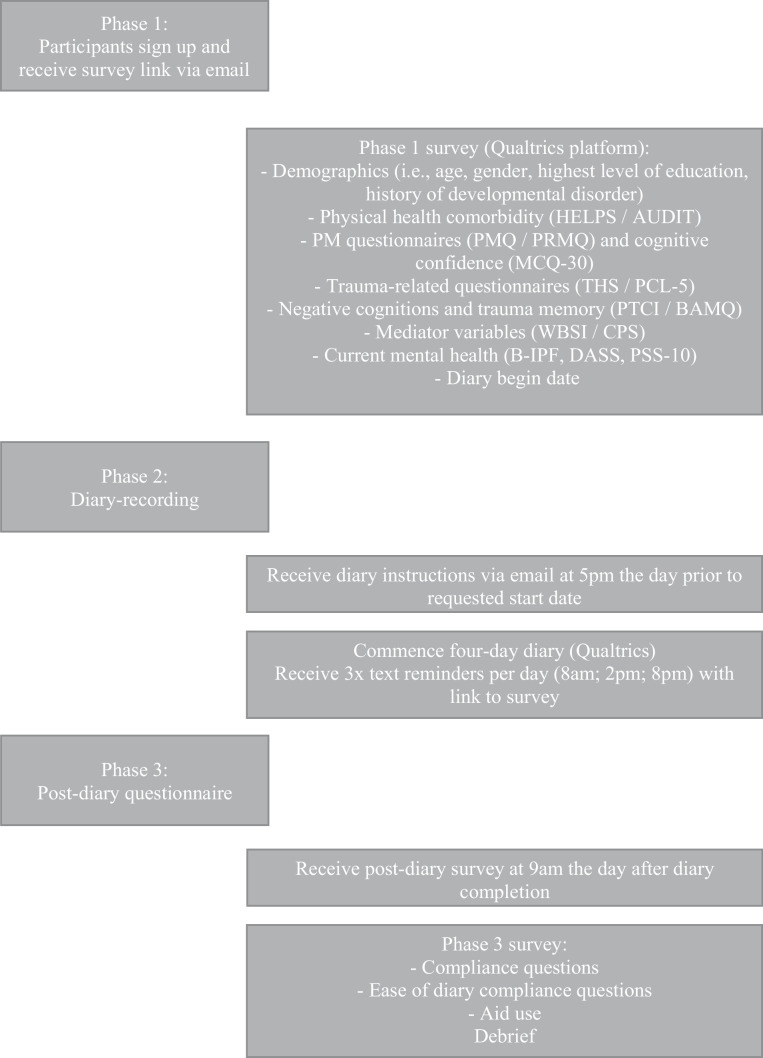
Flow diagram of study procedure

Participation occurred in three phases (see Fig. 1 for a procedure diagram).

##### Phase 1

Participants first completed an online battery of questionnaires (~30–40 min). Participants reported demographics (i.e., age, gender, highest level of education, history of developmental disorder), then responded to the historical/physical health comorbidity questionnaires (i.e., HELPS and AUDIT) in a randomized order. We then presented the prospective memory questionnaires (PMQ, PRMQ) and the cognitive confidence scale (MCQ-30) as one block, in a randomized order. We presented these measures before participants completed questionnaires related to trauma because they are ostensibly unrelated to trauma and reporting a trauma could have influenced scores on these measures. Next, participants completed the trauma-related questionnaires, with trauma history and PTSD symptoms (THS and PCL-5) presented before the negative cognitions about the self and beliefs about trauma memory scales (PTCI, BAMQ in a randomized order). Finally, we presented the remaining mediator variables (i.e., continuous planning, CPS; suppression tendency, WBSI) and current mental health comorbidity variables (i.e., psychosocial functioning, B-IPF; and depression, anxiety and stress, DASS, PSS-10) as a block, in a randomized order. Participants finished by indicating the date they wanted to begin the diary phase within the next 7 days (i.e., Phase 2).

##### Phase 2

Participants received emailed diary instructions at 5 p.m. the day before their requested start date (see OSM for details). On average, participants began the diary 2.07 days after they completed Phase 1. They received the automated text message reminders three times per day (i.e., 8 a.m., 2 p.m. and 8 p.m.), for 4 days. Participants could also self-initiate diary completion any time they realized they made an error, by clicking on the link contained in the reminders.

##### Phase 3

At 9 a.m. on the day after diary completion (i.e., the fifth day), participants received a link to the post-diary survey (~5 min). Participants completed the compliance questions, ease of diary compliance and questions about aid use, respectively.

### Data sorting and analysis

We began data analysis by compiling the list of errors for each participant. A coder (the third author)^2^ checked all errors to ensure that: (1) they met the definition of a PM error (i.e., an action or task to-be-completed in the future at a designated time or in the presence of a specific cue; 132 errors removed), (2) participants had correctly coded their errors as time- or event-based (29 errors changed; two errors not entered as time- or event-based by the participants had enough information for coding), and (3) all recorded errors occurred during the 4-day diary period (19 errors removed). Missing data were minimal in our Phase 1 questionnaires, but were addressed using mean replacement. One participant did not answer enough items from the BAMQ to calculate an accurate total score, so we excluded their score on this scale. One participant completed < 20% of Phase 3, therefore we excluded their data from questions in this phase. For participants’ estimates of the percentage of errors they *recorded*, out of the total errors they *actually* made, responses listing a range (e.g., 50–60%) were changed to the middle of that range to create one data point for analysis (e.g., 55%).

## Results

### Population and diary characteristics

To examine the clinical prevalence of our sample and to explore the descriptive nature of participants’ PM errors we first report descriptive statistics for our self-report scales (Table 1) and diary data. Overall, participants reported subthreshold PTSD symptoms, but 38.5% (*n* = 100) of participants met the cut-off score for probable PTSD (≥ 31; Ashbaugh et al., 2016). Additionally, 31.5% (*n* = 82) of participants were in the severe (11–13) or extremely severe range (14+) for depression, 46.2% (*n* = 120) for anxiety (8–9, 10+, respectively), and 33.8% (*n* = 88) for stress (13–16, 17+, respectively; Lovibond & Lovibond, 1995). On average, participants made 4.82 errors over the 4 days (*SD* = 2.74). We conducted some exploratory *t*-tests that were not pre-registered, finding that participants reported more event- (*M* = 3.59, *SD* = 2.24) than time-based errors (*M* = 1.23, *SD* = 1.36; *t*(259) = -15.31, *p* < .001, *d* = -0.95), and recorded more errors after a reminder (*M* = 2.48, *SD* = 2.04), than freely recalled errors (*M* = 1.80, *SD* = 2.04; *t*(259) = 3.36, *p* < .001, *d* = 0.21). We next explored whether the frequency of diary-recorded errors correlated with self-report PM (see Table 2 for all correlations); there was a small positive correlation for both the PMQ (*r* = .14, 95% CI [0.02, .0.26], *p* = .02) and PRMQ (*r* = .14 [0.02, .0.26], *p* = .02). Time-based errors correlated with the PMQ (*r* = .14, [0.020, .0.26], *p* = .02), but not the PRMQ (*r* = .08, [-0.04, 0.20], *p* = .20); event-based errors correlated with the PRMQ (*r* = .13, [0.01, 0.25], *p* = .04) but not the PMQ (*r* = .09, [-0.03, 0.21], *p* = .17).

**Table 1:** Descriptive statistics for all variables

	Scale	Mean (SD)
PRMQ	8–40	23.09 (6.43)
PMQ	52–260	119.86 (28.13)
PCL Total	0–80	27.18 (18.78)
PCL Re-experiencing	0–20	6.45 (5.23)
PCL Avoidance	0–8	3.52 (2.61)
PCL Alterations in cog/mood	0–12	9.88 (7.31)
PCL Alterations in arousal	0–16	7.33 (6.15)
BAMQ Positive	8–32	14.50 (6.05)
BAMQ Negative	7–28	10.27 (3.69)
MCQ	6–24	12.29 (4.15)
PTCI Self	1–7	2.59 (1.37)
DASS Depression	0–21	7.78 (5.85)
DASS Anxiety	0–21	7.18 (5.50)
DASS Stress	0–21	9.77 (5.52)
CPS	5–20	13.55 (2.46)
WBSI	15–75	53.97 (14.71)
B-IPF	0–48	29.62 (10.41)
PSS-10	0–40	21.56 (6.71)

*PRMQ* Prospective and Retrospective Memory Questionnaire, *PMQ* Prospective Memory Questionnaire, *PCL* Posttraumatic Stress Disorder Checklist, *BAMQ* Beliefs about Memory Questionnaire, *MCQ* Metacognitions Questionnaire, cognitive confidence subscale, *PTCI* self Posttraumatic Cognitions Inventory, negative cognitions about the self subscale, *DASS* Depression, Anxiety and Stress Questionnaire, *CPS* Continuous Planning Scale, *WBS* White Bear Suppression, *B-IPF* Brief Inventory of Psychosocial Functioning, *PSS-10* Perceived Stress Scale

**Table 2:** Correlations between PTSD symptoms, self-report PM, diary-recorded PM and all other comorbidity and mediator variables (A full correlation table between all key and mediator variables appears in our OSM)

	PM err	TB err	EB err	PRMQ	PMQ	PCL Total	PCL B	PCL C	PCL D	PCL E
PM err	-	.58*	.87*	.14*	.14*	.21*	.25*	.18*	.16*	.16*
TB err	-	-	.10	.08	.14*	.29*	.32*	.22*	.23*	.24*
EB err			-	.13*	.09	.08	.11	.09	.05	.05
PRMQ				-	.77*	.34*	.20*	.21*	.33*	.38*
PMQ					-	.34*	.25*	.27*	.31*	.35*
PCL Total						-	.86*	.75*	.92*	.91*
PCL B							-	.67*	.67*	.69*
PCL C								-	.61*	.56*
PCL D									-	.80*
PCL E										-

*PM err* total PM diary-recorded errors, *TB err* time-based PM diary-recorded errors, *EB err* event-based PM diary-recorded errors, *PRMQ* Prospective and Retrospective Memory Questionnaire, *PMQ* Prospective Memory Questionnaire, *PCL* Posttraumatic Stress Disorder Checklist

*p < .05

### PTSD symptoms and prospective memory failures

#### Are PTSD symptoms correlated with everyday diary-recorded PM failures?

Simple correlational analyses on the whole sample (*N* = 260) revealed that total diary-recorded PM failures positively correlated with PTSD symptom severity, a small effect (*r* = .21, 95% CI [0.09, 0.32], *p* < .001).^3^ Consistent with our hypothesis that the relationship would be larger for time- than event-based failures – because time-based tasks rely more on executive functions often impaired in PTSD (e.g., Raskin et al., 2011; Yang et al., 2015) – time-based PM failures correlated with PTSD symptom severity (*r* = .29, [0.18, 0.40], *p* < .001), but event-based PM failures did not (*r* = .08, [-0.04, 0.20], *p* = .18); this difference was statistically significant, *t*(257) = 3.52, *p* = .001. Total failures also correlated with each PTSD symptom subscale (Table 2).^4^

#### Does the relationship between PTSD symptoms and prospective memory arise because of pre-existing vulnerability factors?

We pre-registered to regress diary-recorded PM failures on these variables. However, alcohol dependency (*r* = -.11, 95% CI [-0.012, -.229], *p* = .08) and childhood trauma (*r* = .05, [-0.07, 0.17], *p* = .45) did not correlate with diary-recorded PM failures, therefore we ran the regression controlling only for possible TBI and presence of a learning disorder. We entered possible TBI (*b* = .13, *p* = .04) and presence of a learning disorder (*b* = .18, *p* = .06) simultaneously in Step 1. Together, these variables explained 5.0% variance in diary-recorded PM errors, *R*^*2*^ = .05, *F*(2, 257) = 6.69, *p* = .001. In Step 2, we entered PTSD symptoms (*b* = .16, *p* =.01), which explained a significant additional 2.5% of the variance in diary-recorded failures, *R*^*2*^*change* = .03, *Fchange*(1, 256) = 7.02, *p* =.01. Therefore, even after controlling for physical comorbidities that might account for the PM-PTSD relationship, PTSD symptoms were a significant predictor of diary-recorded PM errors.

#### Do depression, anxiety, and stress contribute to the PM-PTSD relationship?

We pre-registered hierarchical regressions controlling for these variables, and diary-recorded failures. However, depression (*r* = .08, 95% CI [-0.04, 0.20], *p* = .22), and anxiety (*r* = .09, [-0.03, 0.21], *p* = .14) did not correlate with diary-recorded PM errors, therefore we ran the regression only for stress. Stress, over the past week (i.e., DASS stress scores; *b* = .04, *p* = .64) – entered at Step 1 – explained 2.2% of the variance in diary-recorded PM failures, *R*^2^ = .02, *F*(1, 258) = 5.79, *p* = .02. At Step 2, PTSD symptoms explained a small but significant 2.3% of the variance in failures, *R*^*2*^*change* = .02, *Fchange*(1, 257) = 7.21, *p* = .01, *b* = .19. Therefore, PTSD symptoms remained the strongest predictor of diary-recorded PM failures. Given previous research suggests stress might be a key mediator in the PM-PTSD relationship (Swain & Takarangi, 2021, 2022), we pre-registered another hierarchical regression controlling for chronic everyday stress over the past month (i.e., the PSS-10). However, everyday stress did not correlate with diary-recorded PM failures (*r* = .07, [-0.05, 0.19], *p* = .12), so we did not proceed with this analysis.

#### Do PTSD symptoms affect everyday functioning via PM performance?^5^

Psychosocial functioning did *not* correlate with diary-recorded PM failures (*r* = .08, [-0.04, 0.20], *p* = .20), therefore we did not proceed with this analysis.

#### Do metacognitive beliefs and maladaptive strategies mediate the PTSD-PM relationship?

#### Diary-recorded PM

To test the idea – based on previous research (Swain & Takarangi, 2021, 2022) – that the PM-PTSD relationship might be explained via metacognitive beliefs and maladaptive strategies, we planned a series of mediated regressions with diary-recorded errors as the outcome variable. However, diary-recorded PM errors did not correlate with positive beliefs about memory, willingness to future plan, or suppression tendency; therefore, we regressed only cognitive confidence, negative beliefs about memory, and negative cognitions about the self. We used a Bonferroni-corrected alpha of 0.017 to correct for multiple tests (0.05/3). PTSD symptom severity was a significant predictor of diary-recorded PM at Step 1, explaining 4.4% of the variance in errors, *R*^*2*^ = .05, *F*(1, 258) = 11.81, *p* < .001. Beginning with cognitive confidence, at Step 1, this variable explained a non-significant 2.0% of the variance in diary-recorded errors, *R*^*2*^ = .02, *F*(1, 258) = 5.30, *p* =.02, therefore we did not proceed with Step 2. For negative beliefs about memory, at Step 1, this variable explained 4.1% of the variance in diary-recorded errors, *R*^*2*^ = .04, *F*(1, 258) = 11.06, *p* = .001. At Step 2, PTSD symptoms explained a non-significant additional 1.7% variance in diary-recorded errors, *R*^2^*change* = .02, *Fchange*(1, 257) = 4.55, *p* =.03. When entering these variables together, neither beta value was significant in predicting diary-recorded errors (negative beliefs about memory, *b* = .13, *p* = .05; PTSD symptoms, *b* = .15, *p* = .03). For negative cognitions about the self, at Step 1, this variable explained a non-significant 1.7% of the variance in diary-recorded errors, *R*^*2*^ = .02, *F*(1, 258) = 4.40, *p* =.037, therefore we did not proceed with Step 2. Altogether, maladaptive appraisals and strategies did *not* mediate the PM-PTSD relationship when PM was measured via diary-recording.

#### Self-report PM questionnaires

We replicated our findings that negative metacognitive beliefs and maladaptive strategies mediated the relationship between *self-report* PM and PTSD symptoms (see OSM for full statistical analyses). For both the PMQ and the PRMQ, all mediators significantly explained additional variance in scores. Cognitive confidence, negative cognitions about the self and suppression tendency were the most important mediators for *self-report* PM. In summary then, although prior research and our findings here suggest that the *self-report* PM-PTSD relationship is mediated by maladaptive appraisals and strategies (Swain & Takarangi, 2021, 2022), we did not find this relationship for diary-recorded PM errors. In fact, diary-recorded errors correlated only with the self-related variables (i.e., negative cognitions about the self, cognitive confidence). Only negative beliefs about trauma memory mediated the PM-PTSD relationship for diary-recorded PM, but neither these beliefs nor PTSD symptoms uniquely predicted diary-recorded PM failures. Thus, the PM-PTSD relationship when measured via diary-recording is *not* explained by metacognitive beliefs and maladaptive strategies.

### Diary compliance

Because we found that people with worse PTSD symptom severity reported more everyday PM errors, we wondered if they also had poorer compliance on the diary-recording task (i.e., reported recording a lower percentage of forgetting out of ALL their forgetting over the diary-period)^6^ – a PM task. Indeed, people with worse PTSD symptoms estimated they recorded *fewer* errors than they made, indicating poorer compliance with the diary-recording task. Put differently, the percentage of errors participants believed they successfully recorded negatively correlated with PTSD symptoms (*r* = -.19, 95% CI [0.070, 0.31], *p* = .003). To explore whether worse PM performance was attributable to lesser use of memory aids, we correlated the frequency that people reported using aids, with PTSD symptom severity. Aid use in the whole sample did not correlate with PTSD symptoms (*r* = -.05, [-0.07, 0.17], *p* = .39). Of those who *did* report using aids, (i.e., *n* = 111), *frequency* of their aid use was also not correlated with diary-recorded errors (*r* = .07, [-0.05, 0.19], *p* = .47), or PTSD symptoms (*r* = .06, [-0.06, 0.18], *p* = .55).

## Discussion

Overall, our key finding was replication of the PM-PTSD relationship, for both questionnaire-based PM – as measured by the PMQ and the PRMQ – *and* diary-recording; participants who recorded more PM errors over a 4-day period also reported worse PTSD symptom severity. Interestingly, the correlation was significantly smaller for diary-recorded errors (*r* = .21), than for questionnaires (i.e., PMQ and PRMQ; *r*s = .34), *t*(257) = -2.21, *p* = .03. Participants made more event-based than time-based PM errors, but only time-based errors significantly correlated with PTSD symptom severity. *Inconsistent* with prior research (Swain & Takarangi, 2021, 2022), metacognitive beliefs and maladaptive strategies did *not* contribute to the relationship between PTSD symptoms and *diary-recorded* PM, though they continued to explain the relationship with PM *questionnaires*. Thus, perhaps there is a true, albeit small, relationship between PTSD symptoms and everyday PM, but on questionnaires, people’s negative beliefs about themselves and their memory *exaggerate* the size of this relationship.

Our first key finding was that in a general population, PTSD symptom severity correlated with diary-recorded PM errors. This finding aligns with research reporting a PTSD-PM correlation for PM questionnaires, but not for objective, in-lab tasks (i.e., pressing a key after a specified time-period, or when particular words appear). Two lines of thought might explain this pattern. First, perhaps lab-based PM tasks measure a “peak” or “best” performance (e.g., Chaytor & Schmitter-Edgecombe, 2003), rather than typical, everyday performance. Instead, self-report measurement – that captures everyday errors (e.g., forgetting to stop at the shops), and reflects everyday environments, such as *both* questionnaires or diary-recording – may be more representative and generalizable.

Second, both questionnaires and diary-recording likely reflect biased beliefs, in addition to actual PM performance. However, although our analyses both here and in prior research (Swain & Takarangi, 2021, 2022), demonstrate that the PM-PTSD relationship is mediated by maladaptive beliefs and appraisals – specifically cognitive confidence, negative beliefs about memory, suppression tendency and negative cognitions about the self – when using questionnaires, these same beliefs did *not* contribute to diary-recorded errors. Thus, despite diary-recording and questionnaires overlapping in their relevance to everyday PM tasks and task environments, negative metacognitive beliefs seem tied specifically to *PM questionnaires*. An interesting alternative possibility is that diary-recording is biased by metacognitive judgements (e.g., of emotion) that occur in real-time, as participants experience and/or report on their PM errors, rather than by *existing* and more general beliefs, which we measured *before* the diary-period. To explore this possibility, we examined whether participants with worse PTSD symptoms judged the characteristics of their diary-recorded errors – specifically, judgments of their mood, and stress, and the seriousness of their lapses – differently to participants with less severe symptoms. Indeed, participants reporting worse PTSD symptom severity reported more negative mood (*r* = -.15, 95% CI [-0.27, -0.03], *p* = .01) and greater stress (*r* = .28, [0.16, 0.39], *p* < .001), and reported that their errors were more serious (*r* = .22, [0.10, 0.33], *p* < .001). Of course, we cannot verify whether people with worse PTSD symptoms truly experienced higher stress, poorer mood, and/or more serious errors, but we could hypothesize that these people *judged* these experiences as worse in real time. Future research could also manipulate and measure mood, or stress (see Piefke & Glienke, 2017, for review), before a PM task or diary-recording period to examine whether people with worse PTSD symptoms are in a more negative mood, or more stressed, or just *judge* that they are. Thus, it seems likely diary-recording might be biased by real-time metacognitive judgments (e.g., of emotion) – but not by general metacognitive beliefs (e.g., “I am weak”) – instead of reflecting PM performance alone.

Diary-recording might also be biased by participants’ *perception* of their reported errors. Diary-recording requires participants to *notice* the error, *acknowledge* it was an error, and subsequently to *record* that error. In the same way that people with PTSD pay greater attention to, and have enhanced memory for, negative stimuli (e.g., Durand et al., 2019), perhaps for diary-recording, people with worse PTSD symptoms pay more attention to their PM *failures* and therefore report more errors. This increased attention might work as a confirmation bias – seeking or interpreting evidence that confirms pre-existing beliefs/expectations (e.g., Nickerson, 1998). That is, if people with worse PTSD symptoms perceive they are in a more negative mood, or are more stressed, as our data suggest, they might misinterpret or pay greater attention to PM errors that confirm these perceptions. Our data support this idea; people with worse PTSD symptoms estimated recording fewer of their total errors over the diary-period, judging they made a high number of errors, even without evidence. Future research could use a type of cognitive bias modification (e.g., Hoppitt et al., 2010) to educate participants about negative metacognitive biases, to eliminate biases in reporting. Similarly, depression symptoms are another factor that might further contribute to, or exacerbate, negative self-beliefs, and therefore these symptoms should be independently explored in PM research.

Overall, it is possible a small PM-PTSD relationship exists when PM is measured by self-report, but not by lab-based tasks, that are not ecologically valid. Alternatively, it is also possible that lab-based tasks are a “pure” PM assessment PM and both diary-recording and PM questionnaires incorporate metacognitive beliefs and biases in how people perceive their errors.

Our next key finding is that only time- not event-based PM errors correlated with PTSD symptom severity. This finding is consistent with Scott et al. (2016), who found that combat-exposed veterans, regardless of PTSD status, performed similarly on event-based tasks, but those with PTSD demonstrated impaired time-based PM. Yet, other research finds combat-exposed veterans with PTSD perform worse on event-based *and* time-based tasks when compared to non-veteran (Glienke et al., 2017), or non-combat, veteran control groups (Pagulayan et al., 2018) without PTSD. Perhaps combat-stress specifically impairs *both* time- and event-based tasks (e.g., via brain injury or general cognitive deficits), whereas PTSD symptoms impair time-based PM (e.g., due to cognitive resources occupied in symptom management, e.g., Aupperle et al., 2012; Scott et al., 2016). Therefore, trauma type (i.e., combat-stress) likely complicates the PM-PTSD relationship, resulting in inconsistent findings. Given we did not specifically explore trauma type here, we cannot conclude whether trauma type might contribute to the PM-PTSD relationship in a general population. Future research could collect specific trauma types, or control for these experiences in analyses.

The finding that time-based but not event-based failures correlated with PTSD symptom severity provides insight into which PM processes might be impaired amongst people with worse PTSD symptoms. According to the multiprocess framework (McDaniel & Einstein, 2000), PM tasks involve automatic (i.e., bottom-up process involving spontaneous retrieval without ongoing monitoring) or controlled (i.e., top-down memory function involving active maintenance, while scanning for cues; McDaniel & Einstein, 2000; Piefke & Glienke, 2017) cognitive processes. Typically, time-based tasks rely more on controlled processes that rely heavily on executive function (Yang et al., 2015). Thus, we could speculate that these controlled or higher-order executive functioning processes seem impaired in the everyday life of people with PTSD, compared to their automatic counterparts. However, since we did not measure executive function here, we cannot definitively make such a conclusion. Future research should include an executive function measure to explore the possibility that executive function deficits might explain the difference between time- and event-based tasks.

We also found that participants reported more event-based, than time-based, PM errors. In the wider PM literature, time-based tasks are considered more difficult – they require people to monitor and self-initiate performance without external cues (e.g., McDaniel & Einstein, 2000) – than event-based tasks. Therefore, in-lab, event-based PM typically appears superior to time-based performance (e.g., Haines et al., 2020; Jager & Kliegel, 2008). However, recent research suggests this event-based superiority reverses in real-life situations – people perform time-based tasks better (Wójcik et al., 2022). In everyday life, time-based tasks allow for greater aid use – for example, assigning an electronic reminder to take medication at 9 a.m., or for a 4 p.m. meeting. And, time of day (e.g., 9 a.m.) is a more distinct reminder than ambiguous time periods for lab-based tasks (e.g., every 2 min; Kliegel et al., 2008). We also measured only intention *execution*, not planned intentions. Perhaps people complete more everyday event-based – rather than time-based – tasks, leaving greater opportunity for errors, or, were more likely to forget to record, or fail to realize time-based, compared to event-based errors. Thus, future research should aim to measure planned *and* executed intentions (e.g., actual week; Rendell & Craik, 2000).

Our research has limitations. Importantly, the diary task was in itself a PM task. Generally, without a cue to trigger participants they forgot – for example, realizing you forgot to buy milk when making cereal – they might not have realized, and subsequently not recorded such errors. If people with PTSD symptoms have PM difficulties, as our findings suggest, our methodology might not have captured all errors among participants with worse symptoms. To mitigate this issue, we used diary reminders and found that the relationship with PTSD symptom severity and errors recorded after a reminder (*r* = .14, *p* = .02) or after recording a prior error (*r* = .18, *p* = .004) was larger than for freely recalled errors (i.e., not after prompting, *r* = .06, *p* = .37), which was not statistically significant. These results suggest that without prompting, people with worse PTSD symptoms may not have noticed all their errors. Thus, due to using only three reminders per day we may still have underestimated total errors. Additionally, this methodology relied on participants engaging effortfully and understanding definitions we provided (e.g., of PM). To mitigate these issues, we removed participants who recalled less than two errors over the 4 days (Laughland & Kvavilashvili, 2018), and removed errors that did not fit the definition of a PM error. This strategy resulted in removing 132 recorded errors (9.5% of all recorded errors), suggesting some confusion regarding PM definitions. Future research should explore researcher-dictated PM tasks that are consistent with participants’ daily life and priorities, in real-life environments (e.g., actual week; Rendell & Craik, 2000), to preserve experimental control whilst maintaining the representativeness and generalizability of PM tasks, and the experimental environment.

## Conclusions

Overall, our data suggest that questionnaire-based and diary-recorded PM errors – specifically *time-based,* not event-based – correlate with PTSD symptom severity. It seems likely a small correlation exists between everyday PM failures and PTSD symptoms, and this relationship is *exaggerated* on PM questionnaires. This exaggeration might arise from metacognitive beliefs – for example, cognitive confidence – that contribute to the relationship with questionnaires, but not diary-recorded, PM. Therefore, given the different findings based on measurement type, future research should combine different assessment methods to better understand PM impairment for people with PTSD, and, generally, within the PM field. If there is genuine PM impairment among people with PTSD, poor engagement in therapy might result from impaired PM, rather than poor motivation (e.g., nonadherence to homework; Cook et al., 2014; see also Murphy et al., 2002). Thus, psychoeducation or strategies to improve PM, and therapy engagement, would likely enhance treatment outcomes. With continued research we could ultimately assist people with PTSD in attending appointments, taking medication, connecting with social supports, or simply altering negative beliefs, to avoid symptom maintenance or worsening.

## Supplementary information

ESM 1 (DOCX 56 kb)

## Footnotes

1 This research was conducted as part of a larger study and therefore we report only the measures relevant to the current research question(s) here. Participants also reported their mood and stress level immediately prior to the error, and the seriousness of the error. Participants also predicted and postdicted their diary-reported PM performance.

2 The coder identified errors that were not clear, and these errors were examined by a “second coder” (i.e., the first author).

3 As an exploratory analysis that was not pre-registered, we also ran between-groups comparisons on PMQ, PRMQ, total diary-recorded errors, and time- and event-based recorded errors for those above and below the PTSD cut-off. Generally, the results were similar across the PM measures for participants above, and below the cut-off. These full results appear in our OSM.

4 For all following analyses we report only total diary-recorded PM errors as the main outcome variable. Separate analyses for time- and event-based PM appear in our OSM.

5 For the analyses with psychosocial functioning and *self-report* prospective memory, please see OSM.

6 We excluded one person who recorded their percentage as “I have no idea”, thus, *N* = 259.
