# Supplementary material for: I forgot that I forgot: PTSD symptom severity in a general population correlates with everyday diary-recorded prospective memory failures
Source: Mem Cognit. 2023 Feb 22;51(6):1331–45. doi: 10.3758/s13421-023-01400-y (PMC10368574; doi:10.3758/s13421-023-01400-y)
Supplement: Supplementary file 1 — (DOCX 56 kb) [file 13421_2023_1400_MOESM1_ESM.docx]

**Supplementary materials**

**Diary instructions**

Thank you for your participation so far in our study *Forgetting in everyday life.*

As you would be aware, the next phase of the study involves keeping an at-home diary for 4 consecutive days. You are starting your diary XXXXX. The diary for you to complete is **online**and is collected via two methods:

1. You will be reminded at three time points each day via a **text message** to record any forgetting instances you might have had since your last recording. Each text message will include the link to the online diary.
2. You can also self-initiate the recording of your forgetting when you realise either by clicking the **link in this email** (below) yourself. Or by clicking the link in any one of the text messages at any time.

Each time you forget to do a task you had previously planned, we would like you to record the details in this diary. It is important you complete this diary ***as soon as possible***after the forgetting happened, or when you get your closest reminder message. The details you must record include the day, time, what happened, any consequences as a result of your forgetting and some questions about your mood before and after. We would also like you to record whether your instance of forgetting was “time-based” or “event-based”.

Time-based errors involve forgetting to complete a task at a specified time, or after a given period of time. Examples include:

- Forgetting to feed your pet at 6pm
- Forgetting to attend a meeting at 11am
- Forget to take medication every 2 hours
- Forgetting to take your dinner out the oven after 30 minutes

Event-based errors involve forgetting to complete a task when a specific event occurs in your environment, or when a specific cue appears. Examples include:

- Forget to take medication with breakfast
- Forget to turn the heater off when you go to bed
- Forgetting to stop at the shops and pick up milk on your way home
- Forget to message your friend on their birthday

The link to your survey is:  XXXXXX

You can click on this link at any point when you realise you have forgotten to do something, or it will be sent to you at three time points to remind you to complete it.

It’s important you do not change your performance for the purpose of this study. We want an accurate picture of your *typical* forgetting behaviours. That is, avoid using any extra reminders, alarms or lists, just go about your normal behaviour.

At the end of the 4-days you will be sent a final survey to complete.

If you have any questions along the way, please contact XXXX.

**Diary Questions**

**When did you have a memory error?** Date: ___________ Time: __________ AM/PM

**Or when did you realise you made an error?**

**When did you record it here?** Date: ___________ Time: __________ AM/PM

**Describe your memory error:**

**What it was:**

**What you were doing:**

**Where you were:**

**As a reminder:**

Time-based errors involve forgetting to complete a task at a specified time, or after a given period of time. Examples include:

-       Forgetting to feed your pet at 6pm

-       Forgetting to attend a meeting at 11am

-       Forget to take medication every 2 hours

-       Forgetting to take your dinner out the oven after 30 minutes

Event-based errors involve forgetting to complete a task when a specific event occurs in your environment, or when a specific cue appears. Examples include:

-       Forget to take medication with breakfast

-       Forget to turn the heater off when you go to bed

-       Forgetting to stop at the shops and pick up milk on your way home

-       Forget to message your friend on their birthday

**Was the error: time-based 🞏 or event-based 🞏?**

**What was your mood immediately before the error?**

| 🞏 | 🞏 | 🞏 | 🞏 | 🞏 | 🞏 |
| --- | --- | --- | --- | --- | --- |
| Very unhappy |  | Neutral |  | Very happy | Don’t  Know |

**How relaxed or stressed were you immediately before the error?**

| 🞏 | 🞏 | 🞏 | 🞏 | 🞏 | 🞏 |
| --- | --- | --- | --- | --- | --- |
| Very relaxed |  | Neutral |  | Very stressed | Don’t  Know |

**How serious was the memory lapse?**

| 🞏 | 🞏 | 🞏 | 🞏 | 🞏 |
| --- | --- | --- | --- | --- |
| Insignificant | Minor | Somewhat significant | Significant | Very significant / potentially dangerous |

***Correlations between PTSD symptoms, self-report PM, diary-recorded PM and all other comorbidity and mediator variables^[[1]](#footnote-1)^***

|  | PM err | TB err | EB err | PRMQ | PMQ | PCL  tot | PCL B | PCL C | PCL D | PCL E | BAMQ Neg | BAMQ Pos | MCQ | PTCI Self | DASS D | DASS A | DASS S | CPS | WBS | B-IPF | PSS-10 |
| --- | --- | --- | --- | --- | --- | --- | --- | --- | --- | --- | --- | --- | --- | --- | --- | --- | --- | --- | --- | --- | --- |
| PM err | - | .58* | .87* | .14* | .14* | .21* | .25* | .18* | .16* | .16* | .20* | .06 | .14* | .13* | .08 | .09 | .15* | -.03 | .10 | .08 | .07 |
| TB err | - | - | .10 | .08 | .14* | .29* | .32* | .22* | .23* | .24* | .20* | .07 | .11 | .14* | .14* | .14* | .19* | <.001 | .14* | .07 | .12 |
| EB err |  |  | - | .13* | .09 | .08 | .11 | .09 | .05 | .05 | .13* | .03 | .11 | .07 | .01 | .03 | .07 | -.03 | .04 | .05 | .02 |
| PRMQ |  |  |  | - | .77* | .34* | .20* | .21* | .33* | .38* | .20* | .18* | .62* | .38* | .41* | .37* | .42* | .34* | .39* | .40* | .45* |
| PMQ |  |  |  |  | - | .34* | .25* | .27* | .31* | .35* | .27* | .16* | .61* | .30* | .29* | .39* | .39* | .17* | .35* | .28* | .38* |
| PCL Tot |  |  |  |  |  | - | .86* | .75* | .92* | .91* | .47* | .35* | .27* | .64* | .60* | .57* | .60* | .17* | .55* | .46* | .53* |
| PCL B |  |  |  |  |  |  | - | .67* | .67* | .69* | .37* | .29* | .13* | .42* | .40* | .42* | .43* | .04 | .40* | .28* | .37* |
| PCL C |  |  |  |  |  |  |  | - | .61* | .56* | .36* | .30* | .15* | .37* | .37* | .37* | .38* | .08 | .46* | .29* | .38* |
| PCL D |  |  |  |  |  |  |  |  | - | .80* | .46* | .35* | .30* | .68* | .61* | .57* | .57* | .21* | .55* | .46* | .49* |
| PCL E |  |  |  |  |  |  |  |  |  | - | .42* | .29* | .28* | .62* | .61* | .56* | .61* | .20* | .49* | .48* | .55* |
| BAMQ Neg |  |  |  |  |  |  |  |  |  |  | - | .47* | .23* | .54* | .28* | .32* | .33* | .12* | .32* | .27* | .30* |
| BAMQ Pos |  |  |  |  |  |  |  |  |  |  |  | - | .18* | .44* | .27* | .23* | .23* | .09 | .24* | .23* | .24* |
| MCQ |  |  |  |  |  |  |  |  |  |  |  |  | - | .35* | .35* | .35* | .36* | .23* | .30* | .32* | .36* |
| PTCI Self |  |  |  |  |  |  |  |  |  |  |  |  |  | - | .62* | .53* | .56* | .33* | .56* | .54* | .59* |
| DASS D |  |  |  |  |  |  |  |  |  |  |  |  |  |  | - | .70* | .71* | .38* | .59* | .58* | .70* |
| DASS A |  |  |  |  |  |  |  |  |  |  |  |  |  |  |  | - | .78* | .26* | .57* | .40* | .64* |
| DASS S |  |  |  |  |  |  |  |  |  |  |  |  |  |  |  |  | - | .27* | .59* | .51* | .74* |
| CPS |  |  |  |  |  |  |  |  |  |  |  |  |  |  |  |  |  | - | .28* | .21* | .33* |
| WBS |  |  |  |  |  |  |  |  |  |  |  |  |  |  |  |  |  |  | - | .40* | .66* |
| B-IPF |  |  |  |  |  |  |  |  |  |  |  |  |  |  |  |  |  |  |  | - | .55* |
| PSS-10 |  |  |  |  |  |  |  |  |  |  |  |  |  |  |  |  |  |  |  |  | - |

Note: **p* < .05

PM err = total diary PM errors; TB err = time-based diary PM errors, EB err = event-based diary PM errors; PRMQ = Prospective and Retrospective Memory Questionnaire; PMQ = Prospective Memory Questionnaire; PCL = Posttraumatic Stress Disorder Checklist; BAMQ = Beliefs about Memory Questionnaire; MCQ =Metacognitions Questionnaire; PTCI = Posttraumatic Cognitions Inventory; DASS = Depression, Anxiety and Stress Questionnaire, CPS = Continuous Planning Scale; WBS = White Bear Suppression Inventory; B-IPF = Brief Inventory of Psychosocial Functioning; PSS-10 = Perceived Stress Scale.

**Between groups comparisons on key variables**

To exploratorily investigate the difference between those above, and below the cut-off for a likely PTSD diagnosis (>=31; Ashbaugh et al., 2018), we ran independent samples t-tests on our key prospective memory variables. Those above the cut-off reported significantly higher self-report scores on both the PMQ (*M* = 132.02, *SD* = 29.32), *t*(182.92) = -5.62, *p* < .001, *d* = 0.75, and the PRMQ (*M* = 26.06, *SD* = 6.58), *t*(185.41) = -6.08, *p* < .001, *d* = 0.80, compared to those below the cut-off (PMQ: *M* = 112.26, *SD* = 24.55, PRMQ: *M* = 21.24, *SD* = 5.60). Similarly, participants above the PTSD cut-off score also recorded significantly more total diary errors (*M* = 5.45, *SD* = 2.98), compared to participants below the cut off (*M* = 4.43, *SD* = 2.50), *t*(258) = -2.96, *p* = .002, *d* = 0.38. And, the same was true for time-based diary recorded errors; participants above the PTSD cut-off score recorded significantly more time-based errors (*M* = 1.63, *SD* = 1.57), compared to those below the cut-off score (*M* = 0.98, *SD* = 1.14), *t*(164.18) = -2.85, *p* < .001, *d* = 0.49. However, there was no significant difference for event-based diary-recorded errors. Participants above the PTSD cut-off score recorded making a similar number of event-based errors (*M* = 3.82, *SD* = 2.15), compared to those below (*M* = 3.45, *SD* = 2.28), *t*(258) = -1.30, *p* = .097, *d* = 0.17. These findings are consistent with the correlational analyses that the effect size was larger for self-report PM questionnaires, relative to diary-recorded PM. And, that for diary-recorded PM, only the finding for total, and time-based PM errors was significant, but event-based errors was not.

**Time-based and event-based PM analyses for key variables**

We pre-registered to run our key analyses separately for both time- and event-based diary recorded prospective memory. However, because event-based prospective memory did not correlate with PTSD symptoms (*r* = .08, 95% CI [-0.04, 0.20], *p* = .19) we proceeded only with the analyses for *time-based* diary-recorded prospective memory failures. To address the possibility that the relationship between PTSD symptom severity and prospective memory arises because of pre-existing vulnerability factors, we pre-registered to regress *time-based* diary-recorded prospective memory failures on these variables. However, alcohol dependency (*r* = .02, [-0.01, 0.14], *p* = .79) childhood trauma (*r* = .08, [-0.04, 0.20], *p* = .17), and possible TBI (*r* = .07, [-0.05, 0.19], *p* = .25) did not correlate with *time-based* diary-recorded prospective memory failures, therefore we ran the regression controlling only for possible presence of a learning disorder. We entered presence of a learning disorder (*b* = .09, *p* = .13) in Step 1. This variable explained 2.1% variance in *time-based* diary-recorded prospective memory errors, *R^2^* = .02, *F*(2, 257) = 5.42, *p* = .005. In Step 2, we entered PTSD symptom severity (*b* = .27, *p* < .001), which explained a significant additional 7.0% of the variance in total failures, *R^2^change* = .07, *Fchange*(1, 256) = 19.82, *p* < .001. Therefore, even after controlling for physical comorbidities that might account for the relationship between prospective memory and PTSD symptoms, PTSD symptom severity was a significant predictor of *time-based* diary-recorded prospective memory errors.

Next, to test the idea that depression, anxiety and stress might contribute to the relationship between *time-based* prospective memory and PTSD symptoms, we ran hierarchical regressions controlling for these variables. Depression (*b* = -.08, *p* = .38), anxiety (*b* = -.06, *p* = .54), and stress (*b* = .11, *p* = .30), over the past week—entered at Step 1—explained 3.5% of the variance in *time-based* diary-recorded prospective memory failures, *R*^2^ = .04, *F*(3, 256) = 3.07, *p* = .03. At Step 2, PTSD symptoms explained a significant additional 5.4% of the variance in *time-based* failures, *R^2^change* = .05, *Fchange*(1, 255) = 15.03, *p* < .001, *b* = .31. Therefore, PTSD symptoms remained the strongest predictor of *time-based* diary-recorded prospective memory failures. Previous research (Swain & Takarangi, 2021; 2022) suggested that stress (i.e., over the past week) might be a key mediator in the relationship between prospective memory and PTSD symptoms. To explore whether PTSD symptoms explain unique variance in prospective memory errors when controlling for chronic everyday stress over the past month (i.e., scores on the PSS-10), we pre-registered another hierarchical regression controlling specifically for this variable. However, everyday stress did not correlate with *time-based* diary-recorded prospective memory failures (*r* = .12, [-0.002, 0.24], *p* = .06), so we did not proceed with this analysis.

As a secondary interest, we wanted to explore whether PTSD symptoms affect everyday functioning via prospective memory performance. However, psychosocial functioning did *not* correlate with *time-based* diary-recorded prospective memory failures (*r* = .07, [-0.05, 0.19], *p* = .23), therefore we did not proceed with this analysis.

To test the idea—based on previous research (Swain & Takarangi, 2021; 2022)—that the relationship between PTSD and prospective memory might be explained via metacognitive beliefs and maladaptive strategies, we planned to run a series of mediated regressions with *time-based* diary-recorded errors as the outcome variable. However, *time-based* diary-recorded prospective memory errors did not correlate with cognitive confidence, positive beliefs about memory or tendency to future plan therefore we continued the regression controlling only for negative beliefs about memory, negative cognitions about the self and suppression tendency. We used a Bonferroni corrected alpha of 0.017 to correct for multiple tests (0.05/3). PTSD symptom severity was a significant predictor of *time-based* diary-recorded prospective memory at Step 1, explaining 8.3% of the variance in errors, R^2^ = .08, F(1, 258) = 23.20, p < .001. At Step 2, negative beliefs about memory (0.5% variance, *R*^2^*change* = .005, *Fchange*(1, 257) = 1.29, *p* =.26, *b* = .08), negative cognitions about the self (0.3% variance, *R*^2^*change* = .003, *Fchange*(1, 257) = 0.88, *p* = .35, *b* = -.07), and suppression tendency (<0.01% variance, *R*^2^*change* < .001, *Fchange*(1, 257) = .09, *p* = .76, *b* = -.02) did not explain significant additional variance.

**Self-report PM, PTSD and mediator variables**

To test the idea that the relationship between PTSD and self-report PM might be explained by PTSD causing maladaptive appraisals and strategies, and to replicate the finding that negative metacognitive beliefs and maladaptive strategies mediated the relationship between *self-report* prospective memory and PTSD symptoms. we ran a series of mediated regressions. We used a Bonferroni corrected alpha of 0.005 to correct for multiple tests (0.05/5). For the PMQ, PTSD symptom severity was a significant predictor of scores, explaining 11.7% of the variance in PMQ scores, R^2^ = .12, F(1, 258) = 34.12, p < .001 (refer to Table 1 for all statistics). Beginning with cognitive confidence, at Step 1, this variable explained 37.3% of the variance in PMQ scores, but at Step 2, PTSD symptoms explained an additional 3.5% of the variance in PMQ scores. However, based on the beta values, cognitive confidence was a greater predictor of scores than PTSD symptoms. For beliefs about memory, these variables explained 7.3% of the variance in PMQ scores in Step 1. At Step 2, PTSD symptom severity explained an additional 5.8% variance in scores. Based on the beta values, PTSD symptom severity was a better predictor of PMQ scores than were positive and negative beliefs about memory. For negative cognitions about the self, at Step 1, these beliefs explained 9.3% of the variance in PMQ scores. At Step 2, PTSD symptom severity explained an additional 3.6% variance in PMQ scores. Based on the beta scores, PTSD symptoms were a better predictor of PMQ scores than were negative beliefs about the self. For willingness to future plan, at Step 1, this variable explained 2.8% of the variance in PMQ scores. At Step 2, PTSD symptom severity explained an additional 10.1% variance in PMQ scores. Based on the beta scores, PTSD symptoms were a greater predictor of PMQ scores than willingness to future plan. Finally, for suppression tendency, at Step 1, this variable explained 12.1% of the variance in PMQ scores. At Step 2, PTSD symptom severity explained an additional 3.3% variance in PMQ scores. Based on the beta scores, suppression tendency and PTSD symptoms were similar predictors of PMQ scores.

Table 1: Inferential statistics from regression analyses on PTSD and PMQ with maladaptive strategies and negative appraisals as mediators

| Cognitive confidence | *b* | Variance explained (%) | *R^2^* | *R^2^*_change_ | *F* | *F*_change_ | *df* | *p* |
| --- | --- | --- | --- | --- | --- | --- | --- | --- |
| 1. MCQ-30 | .61 | 37.3 | .37 |  | 153.38 |  | 1, 258 | < .001 |
| 1. MCQ-30   PCL-5 (PTSD symptoms) | .56  .19 | 40.8 |  | .04 |  | 15.09 | 1, 257 | < .001 |
| Beliefs about memory |  |  |  |  |  |  |  |  |
| 1. BAMQ - Positive   BAMQ - Negative | .05  .25 | 7.3 | .07 |  | 10.18 |  | 2, 257 | < .001 |
| 1. BAMQ - Positive   BAMQ - Negative  PCL-5 (PTSD symptoms) | -.003  .14  .28 | 13.2 |  | .06 |  | 17.17 | 1, 256 | < .001 |
| Negative cognitions about the self |  |  |  |  |  |  |  |  |
| 1. PTCI – Negative cognitions about the self | .30 | 9.3 | .09 |  | 26.22 |  | 1, 257 | < .001 |
| 1. PTCI – Negative cognitions about the self   PCL-5 (PTSD symptoms) | .15  .25 | 12.8 |  | .04 |  | 10.47 | 1, 256 | .001 |
| Willingness to future plan |  |  |  |  |  |  |  |  |
| 1. CPS | .17 | 2.8 | .03 |  | 7.49 |  | 1, 258 | .007 |
| 1. CPS   PCL-5 (PTSD symptoms) | .11  .32 | 12.9 |  | .10 |  | 29.84 | 1, 257 | < .001 |
| Suppression tendency |  |  |  |  |  |  |  |  |
| 1. WBSI | .35 | 12.1 | .12 |  | 35.56 |  | 1, 258 | < .001 |
| 1. WBSI   PCL-5 (PTSD symptoms) | .23  .22 | 15.4 |  | .03 |  | 9.88 | 1, 257 | .002 |

Note: numbers refer to the step of the hierarchical regression (i.e., 1. Means step 1, and 2. Means step 2).

PCL-5 = Posttraumatic Stress Disorder Checklist; BAMQ = Beliefs about Memory Questionnaire; MCQ = Metacognitions Questionnaire; cognitive confidence subscale; PTCI self = Posttraumatic Cognitions Inventory; negative cognitions about the self subscale; DASS = Depression, Anxiety and Stress Questionnaire, CPS = Continuous Planning Scale; WBS = White Bear Suppression

For the PRMQ, PTSD symptom severity was a significant predictor of PM, explaining 11.5% of the variance in PRMQ scores, R^2^ = .12, F(1, 258) = 33.67, p < .001 (refer to Table 2 for all statistics). Beginning with cognitive confidence, at Step 1, this variable explained 38.2% of the variance in PRMQ scores, but at Step 2, PTSD symptoms explained an additional 3.3% of the variance in PRMQ scores. Based on the beta values, and consistent with results for the PMQ, cognitive confidence was a greater predictor of PRMQ scores than PTSD symptoms. For beliefs about memory, these variables explained 4.9% of the variance in PRMQ scores in Step 1. At Step 2, PTSD symptom severity explained an additional 7.1% variance in scores. Based on the beta values, PTSD symptom severity was a better predictor of PRMQ scores than were negative beliefs about memory. For negative cognitions about the self, at Step 1, these beliefs explained 14.3% of the variance in PRMQ scores. At Step 2, PTSD symptom severity explained a small but additional 1.6% variance in PRMQ scores. Based on the beta scores, negative cognitions about the self were a better predictor of PRMQ scores than PTSD symptoms. For willingness to future plan, at Step 1, this variable explained 11.7% of the variance in PRMQ scores. At Step 2, PTSD symptom severity explained an additional 8.2% variance in PRMQ scores. Based on the beta scores, PTSD symptoms and willingness to future plan were equal predictors of PRMQ scores. Finally for suppression tendency, at Step 1, this variable explained 14.9% of the variance in PRMQ scores. At Step 2, PTSD symptom severity explained an additional 2.3% variance in PRMQ scores. And, suppression tendency was actually a greater predictor of PRMQ scores than PTSD symptoms.

Table 2: Inferential statistics from regression analyses on PTSD and PRMQ with maladaptive strategies and negative appraisals as mediators

| Cognitive confidence | *b* | Variance explained (%) | *R^2^* | *R^2^*_change_ | *F* | *F*_change_ | *df* | *p* |
| --- | --- | --- | --- | --- | --- | --- | --- | --- |
| 1. MCQ-30 | .62 | 38.2 | .38 |  | 159.15 |  | 1, 258 | < .001 |
| 1. MCQ-30   PCL-5 (PTSD symptoms) | .57  .19 | 41.5 |  | .03 |  | 14.63 | 1, 257 | < .001 |
| Beliefs about memory |  |  |  |  |  |  |  |  |
| 1. BAMQ - Positive   BAMQ - Negative | .11  .14 | 4.9 | .05 |  | 6.59 |  | 2, 257 | .002 |
| 1. BAMQ - Positive   BAMQ - Negative  PCL-5 (PTSD symptoms) | .06  .02  .31 | 12.0 |  | .07 |  | 120.73 | 1, 256 | < .001 |
| Negative cognitions about the self |  |  |  |  |  |  |  |  |
| 1. PTCI – Negative cognitions about the self | .38 | 14.3 | .14 |  | 43.00 |  | 1, 257 | < .001 |
| 1. PTCI – Negative cognitions about the self   PCL-5 (PTSD symptoms) | .27  .16 | 15.9 |  | .02 |  | 4.81 | 1, 256 | .029 |
| Willingness to future plan |  |  |  |  |  |  |  |  |
| 1. CPS | .34 | 11.7 | .12 |  | 34.07 |  | 1, 258 | < .001 |
| 1. CPS   PCL-5 (PTSD symptoms) | .29  .29 | 19.9 |  | .08 |  | 26.27 | 1, 257 | < .001 |
| Suppression tendency |  |  |  |  |  |  |  |  |
| 1. WBSI | .39 | 14.9 | .15 |  | 45.33 |  | 1, 258 | < .001 |
| 1. WBSI   PCL-5 (PTSD symptoms) | .29  .18 | 17.3 |  | .02 |  | 7.25 | 1, 257 | .008 |

Note: numbers refer to the step of the hierarchical regression (i.e., 1. Means step 1, and 2. Means step 2).

PCL-5 = Posttraumatic Stress Disorder Checklist; BAMQ = Beliefs about Memory Questionnaire; MCQ = Metacognitions Questionnaire; cognitive confidence subscale; PTCI self = Posttraumatic Cognitions Inventory; negative cognitions about the self subscale; DASS = Depression, Anxiety and Stress Questionnaire, CPS = Continuous Planning Scale; WBS = White Bear Suppression

**Self-report PM, PTSD and psychosocial functioning**

Regarding our secondary interest in psychosocial functioning, we repeated correlational and regression analyses but with *self-report* prospective memory as the outcome variable. Impairment did correlate with self-report prospective memory (PMQ: *r* = .14, 95% CI [0.020, .0.26], *p* = .02, PRMQ: *r* = .14, [0.020, .0.26], *p* = .02). Therefore, we ran a mediated regression to explore whether PTSD symptoms contribute to greater prospective memory errors, which then result in greater everyday impairment. At Step 1, PTSD symptom severity explained 20.7% of the variance in functional impairment scores, *R*^2^ = .21, *F*(1, 258) = 67.24, *p* < .001, *b* = .41. At Step 2, PMQ scores (*b* = .14, *p* = .02) uniquely predicted impairment and explained a small but additional 1.7% of the variance in everyday impairment, *R^2^change* = .02, *Fchange*(1, 257) = 5.63, *p* = .02. Similarly for the PRMQ, at Step 2, PRMQ scores explained an additional 6.7% variance in functioning, *R*^2^*change* = .07, *Fchange*(1, 257) = 23.80, *p* < .001. As for the PMQ, at Step 2, PRMQ scores (*b* = .28, *p* < .001) were a lesser predictor of impairment than PTSD symptoms (*b* = .36, *p* < .001). Therefore, although self-report prospective memory seems related to self-report impairment, PTSD symptom severity remained a greater predictor of impairment scores.

1. Statistical corrections were not applied to these correlations due to them being peripheral to our key research questions. [↑](#footnote-ref-1)
